# Perceived parental prejudice and a tolerant class context in ethnic bullying: The buffering role of teachers

**DOI:** 10.1002/jad.12437

**Published:** 2024-10-22

**Authors:** Maria Chiara Taiti, Benedetta Emanuela Palladino, Federica Stefanelli, Sevgi Bayram Özdemir, Ersilia Menesini

**Affiliations:** ^1^ Department of Education, Languages, Interculture, Literature and Psychology University of Florence Italy; ^2^ School of Law, Psychology and Social Work, Center for Developmental Research Örebro University Sweden

**Keywords:** Classmates’ tolerance, Ethnic bullying, Perceived parental prejudice, Teachers’ tolerance

## Abstract

**Introduction:**

Despite recent efforts to understand the possible impact of contextual factors on adolescents’ involvement in ethnic bullying, most existing studies have focused on the effects of one context at a time. As adolescents are simultaneously exposed to the influence of multiple socialization agents, the aim of this study was to investigate whether teachers’ and classmates’ tolerance towards ethnic minorities could buffer the effect of perceived parental prejudice on adolescents’ involvement in ethnic bullying.

**Methods:**

Data were collected between January and February 2020 from 9th grade adolescents (N = 582; *M*_*age*_ = 15.23; SD = 0.65; 50.9% female; 30.7% with an immigrant background), and their teachers (*N* = 72; aged between 27 and 65 years; 79% female), belonging to 37 classrooms located in Italy.

**Results:**

A cross‐sectional multilevel analysis showed that teachers’ tolerance moderated the effect of perceived parental prejudice on adolescents’ involvement in ethnic bullying. Specifically, we found that in classrooms with low levels of teachers’ tolerance, perceived parental prejudice was significantly associated with students’ involvement in ethnic bullying. Conversely, in classrooms with high levels of teachers’ tolerance, parental prejudice was no longer associated with ethnic bullying. Furthermore, classmates’ tolerance was not significantly associated with students’ involvement in ethnic bullying and did not moderate the association between perceived parental prejudice and adolescents’ engagement in ethnic bullying.

**Conclusions:**

Findings are discussed highlighting the important role of school as a context to promote positive multicultural relations and the unique role played by teachers in affecting adolescents’ behaviors.

## 1 INTRODUCTION

Ethnic bullying has emerged as a significant public concern in schools, particularly in Europe, where an increasing number of children and adolescents belong to ethnic minority communities (IOM, 2019). Ethnic bullying is a form of bias‐based bullying and targets an individual's ethnic background or cultural identity through direct means such as taunts, derogatory comments, or physical aggression, as well as through indirect behaviors such as social exclusion (McKenney et al., 2006). It is sometimes used interchangeably with ethnic discrimination, as both constructs have their roots in prejudice (Taiti et al., 2024). Ethnic bullying includes repeated and intentional threatening or derogatory comments, or exclusionary behaviors, aligning with the traditional definition of bullying (Hellström et al., 2021). Ethnic discrimination, conceptualized as unfair and differential treatment or lack of equal opportunities due to structural and contextual factors (Garcia Coll et al., 1996), does not necessarily need to be repetitive and can be seen as one of the behaviors used by bullies to exclude their peers (Hong et al., 2022).

In the present study, we focused specifically on ethnic bullying but included empirical evidence from previous literature on ethnic discrimination where appropriate.

### 1.1 The role of proximal socialization agents in youth's involvement in ethnic bullying

Adolescents are surrounded by a variety of social contexts and are exposed to the influence of multiple socialization agents, including their parents, classmates, and teachers. While the influence of all these socialization agents on youth attitudes towards minorities is well addressed in the scientific literature (e.g., Bobba et al., 2024; Degner & Dalege, 2013), research on their effects on youth involvement in ethnic bullying is currently scarce. Similarly, there is a lack of studies examining the interaction between the effects of multiple socialization contexts. In the following sections, we review the literature on each of these socialization agents, with a focus on their role in youths’ involvement in ethnic bullying.

#### 1.1.1 The role of parents

Parents and primary caregivers play a crucial role in shaping their offspring's intergroup prejudice development (Bigler & Liben, 2007). Studies have shown that the first signs of intergroup prejudice can be observed at an early age (Raabe & Beelmann, 2011) and that parents and their offspring often share similar attitudes towards people from ethnic minority groups (Degner & Dalege, 2013; Zagrean et al., 2022). This influence is maintained over time from childhood to young adulthood, with the highest levels of parent‐adolescent agreement observed in early adolescence (Gniewosz & Noack, 2015). According to a recent study (Bobba et al., 2024), parents’ stereotypes and beliefs about people from an ethnic minority (i.e., the cognitive dimension of prejudice) longitudinally predict adolescents’ stereotypes and beliefs. In contrast, parents’ negative feelings and dislike towards ethnic minorities (i.e., the affective dimension of prejudice) do not longitudinally predict adolescents’ negative feelings and dislike. If parents’ prejudice influences their children's attitudes towards diversity (e.g., Miklikowska, 2017), we can assume that a behavioral manifestation of such attitudes may also occur in adolescents’ interethnic relations. In other words, perceived parental prejudice towards minorities may be positively correlated with adolescents’ involvement in ethnic bullying. However, to date, no studies have analyzed this association.

#### 1.1.2 The role of teachers

Teachers play a significant role not only as educators but also as socializing agents. They have the ability to foster healthy relationships between students and to prevent negative interactions (Smith et al., 2004). To promote multicultural education, where ethnic discrimination is considered morally unacceptable (Verkuyten & Thijs, 2013), teachers are required to “teach by example” through their own behavior, especially in their relationships with students from different ethnic backgrounds (Geerlings et al., 2019). Teachers’ attitudes toward diversity and their behavior towards people from ethnic minorities have a direct impact on their students’ social experiences within peer groups (McAuliffe et al., 2009). Indeed, they are thought to influence individuals’ behaviors both directly (e.g., what teachers do when a bullying episode occurs; Wachs et al., 2019) and indirectly through the social norms they establish in their classrooms (Glock & Klapproth, 2017). Therefore, as teachers’ attitudes towards ethnic diversity have been found to influence their students’ attitudes (Verkuyten & Thijs, 2013), it is reasonable to hypothesize that such attitudes may also play a role in influencing student's behavior (i.e., ethnic bullying). However, to date, no studies appear to have investigated this relationship.

#### 1.1.3 The role of classmates

Classmates’ attitudes towards minorities may contribute to adolescents’ behaviors in intergroup relationship. Previous studies have shown that adolescents who are surrounded by prejudiced friends, are more likely to behave aggressively towards their immigrant peers and engage in ethnic bullying behaviors (Bayram Özdemir et al., 2016; Bayram Özdemir et al., 2018). In this regard, a recent meta‐analysis highlights that negative attitude among classmates, combined with individual prejudice, are equally important in explaining ethnic bullying among adolescents (Taiti et al., 2024). Although the evidence in this area is still scarce and unclear, structural factors within the classroom also appear to have an impact on classmates’ attitudes and behaviors towards minorities. On the one hand, greater ethnic diversity in the classroom tends to correlate with increased interethnic intolerance among students (e.g., Bayram Özdemir et al., 2018). On the other hand, the literature (e.g., Allport, 1954) emphasizes that ethnic diversity in classrooms can, under certain circumstances, increase tolerance towards minorities and reduce ethnic bullying by facilitating interethnic contact (Graham, 2006). A recent systematic review sought to explain under what circumstances classroom diversity is a protective factor rather than a risk factor for ethnic bullying. According to these findings, ethnic diversity seems to be a risk factor for victimization in the European context (Basilici et al., 2022). Such findings seem to suggest that there is still a need to investigate the link between classmates’ attitudes towards ethnic minorities and students’ behaviors (i.e., ethnic bullying), especially in countries like Italy where the classroom is a very important socialization context.

### 1.2 The current study

Many of the factors that contribute to bullying behavior stem from interactions across different contexts. Research shows that adolescents’ attitudes are significantly influenced by parents’ (e.g., Miklikowska, 2016), teachers’ (e.g., Glock & Klapproth, 2017), and classmates’ (e.g., Bayram Özdemir et al., 2018) negative attitudes towards ethnic minorities. However, less is known about their impact on aggressive behavior. The present study aims to examine the possible interplay among the effects of three socializing agents (i.e., parents, teachers, and classmates) on adolescents’ ethnic bullying behavior, controlling for adolescents’ negative attitudes (i.e., xenophobia), their ethnic background, and the ethnic diversity of the classroom.

Given that bullying often occurs within the school environment, we expect that both classmates’ and teachers’ tolerance towards ethnic diversity could mitigate the association between students perceived parental prejudice and ethnic bullying involvement. Specifically, we hypothesize that when a classroom is characterized by high levels of tolerance towards ethnic minorities among classmates and teachers, the effect of perceived family prejudice on ethnic bullying will be reduced. Furthermore, drawing from the existing literature, we expect that both teachers’ and classmates’ tolerance towards ethnic minorities will have a direct negative impact on ethnic bullying.

## 2 METHOD

### 2.1 Study design and participant recruitment

A cross‐sectional study was conducted from the data collected between January and February 2020, according to the wider research project “*Prejudicial bullying involving ethnic groups: Understanding mechanisms and translating knowledge into effective interventions*” (National PRIN grant 2017 n. 20173E3Z7W _003), funded by the Italian Ministry of Education. University and Research approved by the Ethics Committee of the University of the Sacred Heart of Milan.

Schools were recruited to participate in the project on a voluntary basis through an online invitation. Specifically, the invitation, which requested the participation of at least two 9th grade classrooms, was sent to all high schools in four Italian provinces in Tuscany: Lucca, Florence, Prato, and Pistoia. 13 schools responded to the invitation.

To participate, schools had to obtain prior approval from the school principal and the classroom council. Information letters explaining the study were sent to the students, their parents, and teachers of the participating classrooms. Written consent to participate in the study was obtained from both the parents (or legal representatives) of the students, and from the participating teachers. Before administering the questionnaire, participants (i.e., students and teachers) were informed of the purpose of the research, the methods of data collection, and their right to withdraw from the study at any time without explanation or justification. It was also clarified that participants’ responses would remain anonymous and that data would be aggregated. Data collection took place during regular school hours. The measures were administered using online questionnaires, with a private link provided by the researchers. The paper and pencil version of the questionnaire was handed to those who were unable to complete it online due to various circumstances (e.g., loss of connection or not being issued with a personal mobile phone). After collecting the students’ data, the researchers provided the classroom teachers with the informed consent form. Those who agreed to participate in the study were asked to email the signed form to the researchers. Upon receipt of the module, the researchers responded by sending them the link to the online questionnaire.

In Italy, the classroom functions as the primary formal group for peer socialization. Throughout the 5‐years of upper secondary education, students remain in the same classroom with the same classmates, sharing subjects according to the class timetable. Teachers remain largely the same throughout a student's secondary education. Depending on the subject or subjects they teach, teachers allocate a specific number of hours per week to their students. While the number of hours per week for each subject varies according to the type of school (vocational or classical), teachers of subjects such as mathematics or literature usually spend more time with students than teachers of other subjects, such as physical education or religious education.

### 2.2 Participants

Initially, 58 classrooms were recruited from 13 secondary schools located in Tuscany. The current study focused on students and teachers coming from the same classrooms. Thus, 21 classrooms were excluded from the analysis due to missing teachers’ data. The analytical sample of the current study included data from students and teachers from 37 classrooms in 12 secondary schools.

The students sample comprised 582 adolescents (*M*_*age*_ = 15.23; SD = 0.65; 50.9% girls). Most of them (87.8%) were born in Italy and the rest in Central, Northern and Eastern Europe (4.5%), Asia (1.4%), Africa (1.6%), South America (5.8%) and North America (0.3%). About one‐third of the total sample (30.6%) had at least one parent born abroad. Their parents came from around 50 different countries, including China (6.3%), Albania (6.1%), Romania (3.4%), and Morocco (2.7%). Most participants (98.6%) lived with both their biological parents. A small percentage (0.8%) lived in with other family members (e.g., grandparents, uncles) while 0.5% lived with other people (e.g., adoptive mother). One fourth (20.2%) of the students reported being the only child while 51.8% reported having one sibling. Additionally, 20.4% reported having two siblings, 5.9% reported having three siblings, and 1.7% reported having four or more siblings.

The teachers sample consisted of 72 teachers, with an age range between 27 and 65 years (*M*_*age*_ = 47.66, SD = 10.59; 79.2% females). All of them were born in Italy, except for one teacher who originated from Sri Lanka. In each classroom, the number of teachers who completed the questionnaire ranged from a minimum of 1 to a maximum of 7 (*M* = 2.65, SD = 1.69). The reported number of teaching hours per classroom by these teachers ranged from a minimum of 1 to a maximum of 10 h per week (*M* = 4.63, SD = 2.67).

### 2.3 Measures

#### 2.3.1 Perceived parental prejudice

A revised version of Stephan and colleagues’ scale (Stephan et al., 1999) was used to measure adolescents’ perspective of their parents’ attitudes towards people of different origin or ethnicity (Papotti et al., 2021; Papotti, 2020). To assess students' perceived parental prejudice, adolescents were asked to rate 5 items on a 7‐point Likert scale ranging from 1 (*Strongly agree*) to 7 (*Strongly disagree*). Examples of items are: “In my family it is thought that people of different ethnicity/origin get more than they deserve” or “In my family it is thought that people of different ethnicity/origin are a threat”. Students’ responses to the scale items were averaged to create the scale scores, where higher scores indicated a greater perception of parental prejudice. In the present sample, CFA showed good fit indices, except for Chi Squared *p*, which is especially sensitive to sample size (*χ* ^*2*^_(5)_ = 18.830, *p* = .002; CFI = 0.971; RMSEA = 0.069). Factor loadings ranged from 0.48 to 0.77. Cronbach's alpha, used as an index of internal consistency, showed good reliability of the measure (*α* = .73, 95% CI [.70, 0.77]).

#### 2.3.2 Individual tolerance and xenophobia towards people with an ethnic background

The Tolerance and Xenophobia scale (Van Zalk et al., 2013) was used to assess student's attitudes toward people with a different ethnic background. The items of the original scale were minimally modified (Van Zalk et al., 2013) to be suited in measuring tolerance and xenophobia towards people with a different ethnicity or culture who live in Italy. The scale consists of two subscales: Tolerance (4 items, i.e., “We should have a welcoming attitude toward people with a different ethnicity/origin who live in Italy.”) and Xenophobia (4 items, i.e., “People with a different ethnicity/origin increase criminality”). Students were asked to report the extent to which they agreed or disagreed with the statements on a 4‐point Likert scale ranging from 1 (*Strongly disagree*) to 4 (*Strongly agree*). In the present study, the bifactorial CFA model on students’ data showed good fit indices, except for Chi‐Squared significant test, which is sensitive to sample size (*χ* ^*2*^_(19)_ = 34.426, *p*. = 0.016; CFI = 0.985; RMSEA = 0.038). Students’ responses to the Xenophobia scale items were averaged to create scale scores, with higher scores indicating higher levels of xenophobia towards ethnic minorities (i.e., at the within level in the tested model). Cronbach's alpha demonstrated good reliability for students’ tolerance (*α* = .75, 95% CI [.72, 0.78]) and xenophobia subscales. (*α* = .75, 95% CI [.72, 0.79]).

#### 2.3.3 Classmates’ tolerance towards people with an ethnic background

Conversely, students’ responses to the Tolerance scale items were averaged within each classroom to create classroom scores of tolerance towards ethnic minorities (i.e., between levels). Higher scores indicated high levels of classroom's tolerance.

#### 2.3.4 Teachers’ tolerance towards people with an ethnic background

Teachers were asked to complete the same tolerance subscale as students. The same CFA model as for the students, was run on the teachers’ data and showed good indices of fit (*χ* ^*2*^_(2)_ = 0.154, *p*. = 0.926; CFI = 1.000; RMSEA = 0.000). Cronbach's alpha demonstrated good reliability for the teachers’ tolerance subscale (*α* = .80, 95% CI [.71, 0.87]). As previously mentioned, not all teachers reported teaching the same number of hours, as this varied based on the subject they taught. Based on the hypothesis suggesting that teachers who spend more time in the classroom exert greater influence, we calculated the variable *Teachers’ tolerance* as follows: teacher's score to the tolerance subscale (Van Zalk et al., 2013) was multiplied by the number of hours they reported spending in their classroom. In instances where multiple teachers from a classroom responded to the questionnaire, these scores were averaged to establish a classroom teachers’ tolerance level (i.e., between level). Higher scores indicated high levels of teachers’ tolerance.

#### 2.3.5 Ethnic bullying

An adapted‐revised version (Palladino et al., 2020) of the Florence Victimization and Bullying Scale (Palladino et al., 2016) was used to assess ethnic bullying. The subscale to assess bullying is composed of 4 items asking how often, in the last 3 months, students had physically (i.e., “I have beaten…”), verbally (i.e., “I have made fun of…”), and indirectly (i.e., “I have excluded…”; “I've spread rumors…”) attacked someone “because of his/her culture or country of origin”. Items were rated on a 5‐point Likert scale from 1 (*Never*) to 5 (*Several times a week*). Responses to scale items were averaged to create the scale scores, with higher scores indicating high levels of involvement in ethnic bullying. A definition of bullying introduced the scale (see appendix). Within our data, CFA with maximum likelihood estimation and robust standard errors (MLR) showed good fit indices (*χ* ^*2*^_(2)_ = 5.260, *p*. = 0.072; CFI = 0.944; RMSEA = 0.053), with factor loadings ranging from 0.49 to 0.99. Cronbach's alpha, used as an index of internal consistency, showed good reliability of the measure (*α* = .79, 95% CI [.76, 0.82]).

#### 2.3.6 Ethnic background

To control for students’ ethnic background, we distinguished between students with at least one foreign‐born parent and those with both parents born in Italy. Hence, a dichotomous variable was created where “0” represented students with an ethnic background, and “1” represented the Italian students; 69.3% of the sample were Italian, and 30.7% were students with an ethnic background.

#### 2.3.7 Ethnic diversity

Variable at the class‐level was calculated using the Simpson's Diversity index (1949). This index allowed us to capture both the number of different ethnic groups, and the relative representation of each group in each class. Scores ranged from 0 to 1, with higher numbers reflecting greater ethnic diversity. To calculate the diversity index, we used adolescents’ data on their parents’ country of birth. The average ethnic diversity index for all classes in the present study is 0.47 (SD = 0.25; min = 0; max = 0.87), and the average class‐size is approximately 15 students (SD = 4.85; min= 5 students; max= 24 students).

### 2.4 Data analysis

As a preliminary step, Confirmatory Factor Analyses (CFAs) were conducted on each measure included in the study (i.e., Perceived parental prejudice, Tolerance and Xenophobia scale, and Ethnic bullying) to assess whether our data aligned with the measurement models tested in the original scale validation studies. The models were evaluated using the following fit indices: the chi‐square (χ2) statistic, the root‐mean‐squared error of approximation (RMSEA), and the comparative fit index (CFI). Recommended cut‐off points for these measures are 0.08 (Browne & Cudeck, 1993) or 0.06 (Hu & Bentler, 1999) for RMSEA, 0.90 or 0.95 for CFI (Bollen, 1989). See the Measure section for the results.

A random intercepts multilevel model (Hox et al., 2017; Snijders & Bosker, 2011) with two levels of analysis (level 1: individuals, and level 2: classes) was used to examine the interaction between proximal social contexts and adolescents’ ethnic bullying behaviors. Multilevel procedures deal with complex situations in which experimental units are nested in a hierarchy and allow the exploration of how relationships between variables vary at different levels. Mplus Version 7 (Muthén & Muthén, 2012) was used to perform the model. In addition, to control for the nested nature of our data, we included the classroom variable as a cluster in the multilevel model. As a strategy of analysis, the cross‐level interactions were tested (i.e., teachers’ tolerance X perceived parental prejudice; classmates’ tolerance X perceived parental prejudice) and if they were not significant, they were removed from the final model.

Some of the measured variables were aggregated to create classroom‐level variables. Specifically, classmates’ tolerance levels were averaged within each classroom to create classroom‐level measures. A similar procedure was followed for teachers’ tolerance: the mean score of teachers’ responses to the Tolerance's subscale (Van Zalk et al., 2013) was multiplied by the number of hours each teacher spent in each class to create an averaged measure of teachers’ tolerance. If a classroom had more than one teacher's score, the scores were averaged to create the classroom measure. At the individual level, perceived parental prejudice was used as a focal predictor of ethnic bullying, controlling for students’ ethnic background and students’ individual prejudice, while teachers’ and classmates’ tolerance were the main predictors at the classroom level, controlling for the ethnic diversity of the classroom. Group mean centering was used for all the predictors at the individual level, and grand mean centering was used for all the predictor variables at the classroom level (Enders & Tofighi, 2007). Given the non‐normal distribution of the outcome variable, a maximum likelihood parameter estimator with robust standard errors (MLR) was used to obtain robust estimates (Yuan & Bentler, 2000). We used Little's test analysis (1988) to test whether missing values occurred completely at random (MCAR). Our missing data were MCAR, as indicated by the nonsignificant Little's test (*χ* ^2^_(26)_ = 22.538, *p* = .659). Missing data were therefore treated using full information maximum likelihood (FIML) for their estimation.

## 3 RESULTS

Means, standard deviations, and correlations among the study variables are presented in Table 1.

**Table 1:** Means, Standard Deviations and Bivariate Correlations of the study variables.

Student‐Level Variables	*M*	SD	Min ‐ Max	*N*	1.		2.		3.	
**1.** Student's Xenophobia	2.05	0.67	1–4	582	**‐**					
**2.** Perceived Parental Prejudice	3.34	0.93	1–6.5	582	**0.46 (**<0**.001)**		**‐**			
**3.** Ethnic Bullying	1.05	0.26	1−4.5	582	**0.19 (**<0**.001)**		**0.18 (**<0**.001)**		**‐**	
**Class‐Level Variables**	*M*	SD	Min ‐ Max	*N*	**1.**	**2.**		**3.**		**4.**	
**1.** Ethnic Diversity	0.47	0.25	0–0.87	582	‐						
**2.** Classmates’ Tolerance	3.10	0.23	2.5–3.61	582	− 0.17 (0.309)	‐					
**3.** Teachers’ Tolerance	11.73	5.78	4–37.5	582	**− 0.38 (0.013)**	0.18		‐			
**4.** Ethnic Bullying	1.05	0.10	1–1.62	582	**0.08 (0.030)**	− 0.14 (0.052)		**− 0.05 (0.030)**		‐	

*Note*: *p* ‐values are reported in brackets and significant correlations are shown in bold.

The intraclass correlation coefficient, based on the null model of the multilevel model, showed that 8.6% of the variance in adolescents’ involvement in ethnic bullying was at the classroom level. Multilevel analysis is, therefore, the most appropriate strategy to examine the link between parents’ prejudicial beliefs (controlling for the students’ ethnic background and their xenophobia), classmates’ and teachers’ tolerance (controlling for the ethnic diversity of the class), and adolescents’ involvement in ethnic bullying.

**Figure 1:**
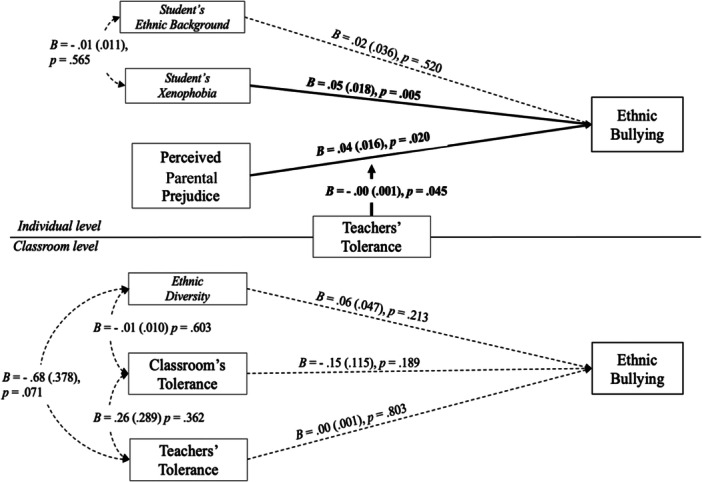
Unstandardized estimates of the final multilevel model. *Note*. Solid line= significant effects (*p* < .05); Dotted line= not significant effects (*p* > .05). Standard errors are reported in brackets and significant unstandardized estimates (*p* < .05) in bold.

The results of the final model (see Figure 1 and Table 2) showed that at the individual level, perceived parental prejudice (*B* = 0.037, *SE* = 0.016, *p* = .020) and student's xenophobia (*B* = 0.052, *SE* = 0.018, *p* = .005) were significantly and positively associated with ethnic bullying. No significant main effects were found at the class level.

**Table 2:** Multilevel mixed model results predicting Ethnic Bullying.

Effects	Est.	SE	*p* ‐ Value
**Fixed effects**			
**Student Level**			
Ethnic Background (0= Immigrant Background, 1= *Blind*)	0.023	.036	.520
Student's Xenophobia	**0.052**	**.018**	**.005**
Perceived Parental Prejudice	**0.037**	**.016**	**.020**
**Class Level**			
Ethnic Diversity	0.059	.047	.213
Classmates’ Tolerance	− 0.151	.115	.189
Teachers’ Tolerance	0.000	.001	.803
**Cross‐level interaction**			
Teachers’ Tolerance X Perceived Parental Prejudice	**− 0.002**	**.001**	**.045**

*Note*: statistically significant results are shown in bold.

Est. = unstandardized estimates; (SE) = standard error of the distribution

**Figure 2:**
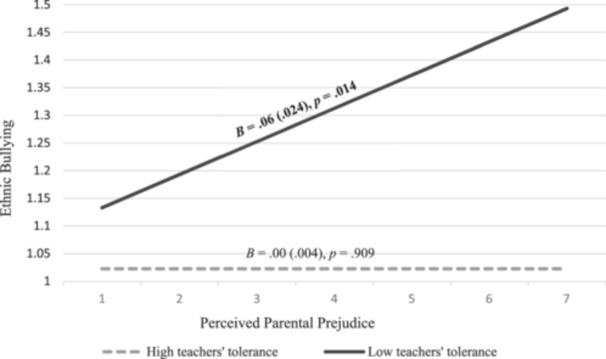
Moderation plot of the cross‐level interaction between Perceived Parental Prejudice and Teachers’ Tolerance on Ethnic Bullying. *Note*. Significant unstandardized estimates (*p* <.05) are reported in bold with the standard error in brackets.

Only the cross‐level interaction between teachers’ tolerance and perceived parental prejudice with ethnic bullying was found to be significant (*B* = ‐ 0.002, *SE* = 0.001, *p* = .045). Specifically, we found that in classes with low levels of teachers’ tolerance (scores below the mean level of teachers’ tolerance, i.e., *M* = 11.73), perceived parental prejudice is significantly associated with adolescents’ ethnic bullying (*B* = 0.060, *SE* = 0.024, *p* = .014). Conversely, in classes with high levels of teachers’ tolerance (scores equal to and above the mean level of teachers’ tolerance), parental prejudice is no longer associated with ethnic bullying (*B* = 0.000, *SE* = 0.004, *p* = .909) (see Figure 2).

## 4 DISCUSSION

Despite recent efforts to understand the implications of contextual effects on adolescents’ attitudes toward ethnic minorities, less is known about the relationship between different socialization agents’ attitudes toward diversity and adolescents’ involvement in ethnic bullying. Furthermore, although the congruence between parents’ and their offspring's attitudes towards ethnic minorities is well documented in the literature, the association between parental prejudice and students’ negative behavior (i.e., ethnic bullying) remains understudied. Finally, the possible buffering effect of a positive, tolerant school environment on aggressive behavior towards immigrant peers has not yet been demonstrated. Therefore, the purpose of the present study was to provide a better understanding of the relationship between parental prejudice and ethnic bullying, and whether a tolerant school context (i.e., teacher and peer tolerance) can mitigate the association between perceived parental prejudice and adolescents’ involvement in ethnic bullying.

First, in addition to the significant direct association of student's individual xenophobia and ethnic bullying, which is consistent with previous literature (e.g., Bayram Özdemir et al., 2016), our study highlights a significant cross‐level interaction between teachers’ tolerant beliefs toward ethnic minorities and perceived parental prejudice towards ethnic minorities on ethnic bullying. When teachers’ tolerance was low, there was a positive association between perceived their parental prejudice and adolescents’ involvement in ethnic bullying. This finding suggests that attending a class where teachers are unwelcoming to ethnic minority students or do not value ethnic diversity, combined with growing up in families with strong prejudices and stereotypes against ethnic minorities, is positively associated with the risk of adolescents engaging in aggressive behavior towards ethnic minority peers. This finding extends previous research on the transmission of negative attitudes from parents to their children (e.g., Miklikowska, 2016, 2017). Indeed, theories of prejudice development suggest that parents are important for the development of prejudice (Allport, 1954; Bigler & Liben, 2007). In this vein, our findings extend the previous literature by providing important insights into the influence of the family context on children's behavior. Parental attitudes not only play a role in influencing their offspring's negative attitudes but also influence their aggressive behavior (i.e., ethnic bullying) when other significant adults (i.e., teachers) hold less tolerant attitudes towards ethnic minority students.

In the Italian school system, the classroom is the primary setting where youths spend most of their daily time together with classmates and teachers. Studies have shown that perceived classroom norms regarding attitudes towards out‐groups (in terms of classmates’ and teachers’ attitudes towards cultural diversity) are related to students’ own attitudes towards out‐groups (Karataş et al., 2022; Thijs & Verkuyten, 2013). Our findings extend previous literature by further highlighting that the influence of parents in explaining ethnic bullying behavior decreases when there is a high level of tolerance in the classroom (i.e., teachers’ positive attitudes towards ethnic minorities). Consequently, teachers’ tolerance towards ethnic minorities acts as a protective factor for students’ involvement in ethnic bullying, even in the presence of prejudiced parents towards ethnic minorities. There are two reasons for this finding. First, teachers are important in shaping classroom norms, which, together with attitudes, are strong predictors of behavior (Fishbein & Ajzen, 1975). Second, teachers play an important role in students’ decisions about social inclusion and exclusion (Juvonen et al., 2019). Their influence extends beyond the mere endorsement of intergroup contact to encompass the interpersonal relationships they endorse with their students. Previous research has shown that teachers’ interactions with students have a strong influence on how relations are formed between students (e.g., Farmer et al., 2019; Mikami et al., 2012) and that they play a protective role in preventing negative interethnic relations (Grütter et al., 2021). The way in which teachers embody multiculturalism in the student–teacher relationship (their attitudes towards minority students) seems to be more relevant than what they verbally proclaim about cultural diversity. In this context, the role of teachers in the formation of adolescents’ attitudes has already been investigated (e.g., Bigler & Liben, 2007; Castelli et al., 2008) and is consistent with the transmission of implicit negative attitudes from teachers to their students (Vezzali et al., 2012).

Our results also showed that classmates’ tolerance was not significantly associated with students’ involvement in ethnic bullying, which contrasts with a recent meta‐analysis (Taiti et al., 2024). Nevertheless, this finding needs to be interpreted considering that the data collection took place only a few months after the beginning of the first year of high school – and, we can assume, only a few months after the students had met each other for the first time. Thus, classroom norms about the inclusion or exclusion of peers from a different ethnicity or culture may not have yet emerged and been established. Indeed, research has shown that group norms predict intergroup interactions, because of the need to belong and the desire to avoid displaying attitudes or behaviors that are incompatible with those of the group (Nesdale, 2004, 2007). A growing body of research has shown that positive contact norms in class (e.g., inclusiveness, mutual respect, cooperative classroom activities) are associated with lower levels of prejudice (Molina & Wittig, 2006) and, in turn, with fewer episodes of ethnic victimization (Bayram Özdemir & Özdemir, 2020). This result opens up interesting future perspectives regarding the possibility of testing the same hypothesis using longitudinal data and in different school years.

In conclusion, these findings emphasize the need to consider interventions aimed at preventing or reducing ethnic bullying within a broader ecological interaction model (Garcìa Coll et al., 1996), in which individual behavior is the result of interactions between variables operating mainly at the microsystem level (e.g., parents and teachers) (Bronfenbrenner, 1979).

### 4.1 Limitations and future directions

These results need to be considered with a few limitations. The first concerns the measurement of teachers’ tolerant attitudes towards ethnic minorities. Given the institutional role of teachers as educators and contextual role models for students, it is plausible that they may have given responses more in line with their perceived standards of social desirability than their true beliefs. In addition, teachers were asked to voluntarily decide whether or not to participate in the data collection, which led to great variability between and within classes. In some classes, there were up to seven informants, while in other classes only one teacher completed the questionnaire. These teachers may only spend a few hours in the class, which limits the possibility to influence the students. To overcome these issues, this measure was weighted by the number of hours each teacher spent in the classroom. However, this approach should be refined in future studies to improve its validity and reliability. Implicit measures, for instance, have shown promising results in assessing teachers’ attitudes and stereotypes, as they are less susceptible to social desirability problems, and can measure associations that one may not be aware of (Denessen et al., 2022).

Secondly, it is essential to consider the implications of measurement issues when interpreting the nonsignificant association between classmates’ tolerance and students’ involvement in ethnic bullying. The prospect of testing the same hypothesis with longitudinal data in different school years is an exciting avenue for future research, particularly given that the early timing of data collection may not have allowed for the development of classroom norms regarding the inclusion or exclusion of peers from different ethnic or cultural backgrounds.

One dimension we have not explored is the potential role of other processes in moderating the association between prejudice and behavior among minority and majority groups. Indeed, previous studies have shown that the level of acculturation may moderate the effects of ethnicity. Research suggests that second‐generation immigrants have more similar perceptions of the school environment than first‐generation immigrants (Phalet & Andriessen, 2003; Ward & Kennedy, 1993); future studies should further explore these differences between subgroups of the population. In addition, the decision to limit reporting of prejudice and tolerance towards people whose ethnicity differs from that of a particular country (i.e., Italy) may have hindered the identification of other forms of intolerance, such as the attitudes of minority versus majority members. It would be beneficial for future studies to consider using an alternative scale to accurately capture the full spectrum of intergroup attitudes.

Furthermore, the variable of parental prejudice is a self‐reported measure of the students’ perceptions. This methodological aspect limits the research in the sense that it may not be representative of the investigation of parental attitudes towards ethnic minorities, but the result of students’ perceptions. Future studies may wish to replicate our findings using multi‐informant measures, including parent‐reported data on parental prejudice. Given the potential biases associated with self‐reported data, such an approach may allow for more robust conclusions (Yu, 2010). Finally, as noted above, it will be beneficial to use longitudinal data collection to better capture the developmental changes in students’ proximal contextual influences (i.e., classmates). With these considerations in mind, we draw the following conclusions.

## 5 CONCLUSIONS

Our study has demonstrated that during adolescence, students’ involvement in ethnic bullying is influenced by prejudice perceived within the family context (i.e., parents), especially when teachers have low levels of tolerance towards ethnic minorities. This finding highlights the crucial role of teachers in buffering the potential negative effects of parental prejudice on adolescents’ behaviors in diverse peer contexts and suggests that future research should prioritize the school context for interventions aimed at reducing ethnic bullying. Increasing attention has been paid to the role of teachers in shaping the context of positive interethnic relations in classrooms (Grütter et al., 2021; Iannello et al., 2021), and looking at our results, it seems to be crucial in the early years of high school. Indeed, teachers’ beliefs about ethnic diversity and their adherence to diversity‐related norms provide fertile ground for intervention, both through awareness‐raising initiatives and by acting as positive role models. Our findings underscore the importance of considering these factors when designing tailored programs aimed at reducing such negative behaviors. Multicomponent interventions that emphasize the role of teachers may hold promise for effectively reducing the risks of ethnic bullying and discrimination among adolescents, even in contexts where family attitudes towards ethnic minorities are unfavorable.

### 1 CONFLICTS OF INTEREST STATEMENT

The authors declare no conflicts of interest.

## Data Availability

The data that support the findings of this study are available on request from the corresponding author. The data are not publicly available due to privacy or ethical restrictions.
